# Vicarious pain while observing another in pain: an experimental approach

**DOI:** 10.3389/fnhum.2013.00265

**Published:** 2013-06-11

**Authors:** S. Vandenbroucke, G. Crombez, D. M. L. Van Ryckeghem, M. Brass, S. Van Damme, L. Goubert

**Affiliations:** ^1^Department of Experimental-Clinical and Health Psychology, Ghent UniversityGhent, Belgium; ^2^Department of Experimental Psychology, Ghent UniversityGhent, Belgium

**Keywords:** vicarious pain, synaesthesia for pain, observation of pain, empathy, hypervigilance for pain

## Abstract

**Objective:** This study aimed at developing an experimental paradigm to assess vicarious pain experiences. We further explored the putative moderating role of observer's characteristics such as hypervigilance for pain and dispositional empathy.

**Methods:** Two experiments are reported using a similar procedure. Undergraduate students were selected based upon whether they reported vicarious pain in daily life, and categorized into a pain responder group or a comparison group. Participants were presented a series of videos showing hands being pricked whilst receiving occasionally pricking (electrocutaneous) stimuli themselves. In congruent trials, pricking and visual stimuli were applied to the same spatial location. In incongruent trials, pricking and visual stimuli were in the opposite spatial location. Participants were required to report on which location they felt a pricking sensation. Of primary interest was the effect of viewing another in pain upon vicarious pain errors, i.e., the number of trials in which an illusionary sensation was reported. Furthermore, we explored the effect of individual differences in hypervigilance to pain, dispositional empathy and the rubber hand illusion (RHI) upon vicarious pain errors.

**Results:** Results of both experiments indicated that the number of vicarious pain errors was overall low. In line with expectations, the number of vicarious pain errors was higher in the pain responder group than in the comparison group. Self-reported hypervigilance for pain lowered the probability of reporting vicarious pain errors in the pain responder group, but dispositional empathy and the RHI did not.

**Conclusion:** Our paradigm allows measuring vicarious pain experiences in students. However, the prevalence of vicarious experiences of pain is low, and only a small percentage of participants display the phenomenon. It remains however unknown which variables affect its occurrence.

## General introduction

Viewing someone in pain has been suggested to elicit distress in observers (Goubert et al., 2005, 2009). In addition, several brain regions tapping into the affective-motivational properties of pain have been found to become activated when seeing someone else in pain (Jackson et al., 2005). Furthermore, studies have provided evidence that observing others' pain activates brain regions subserving the sensory-discriminative properties of pain (Bufalari et al., 2007). Intriguingly, observing pain in others may also give rise to a vicarious experience of pain. This experience has most often been described in patients with a history of intense, traumatic pain. For example, Giummarra and Bradshaw (2008) documented a case of vicarious pain in a woman who had an emergency caesarean section delivery because of a long and painful labor with obstruction. This woman reported the experience of “shooting pains from the groin that radiate down the legs” when told of another's traumatic experience. In another study with 74 phantom limb patients (Fitzgibbon et al., 2010a), 16% of the participants reported that observing or imagining pain in another person triggers their phantom pain. There is little research yet available on the occurrence of vicarious pain and underlying mechanisms (but see Fitzgibbon et al., 2012a,b). Most evidence stems from clinical studies, using self-report questionnaires, describing the phenomenon and research in amputees. Little is known whether vicarious pain experiences can be elicited in a more systematic way, for example by means of an experimental paradigm in a lab.

There is preliminary evidence that also individuals without traumatic pain experiences may feel pain by observing pain in others. Osborn and Derbyshire (2010) found that, when healthy volunteers were presented a series of images and video clips depicting painful events, almost 30% reported at least one pain experience. In a follow-up study, 10 of these vicarious pain responders were matched with 10 non-responders to take part in an fMRI study, and static images of painful events and emotional images not containing noxious events were shown. When observing the images of the painful events, vicarious pain responders showed higher activation of emotional (i.e., left and right insular) and sensory brain regions (i.e., secondary somatosensory cortex) associated with pain than non-responders.

The mechanisms and conditions that affect these vicarious experiences are largely unknown. Fitzgibbon and colleagues (2010b) proposed a framework to further our understanding of vicarious pain, which they dubbed “synesthesia for pain.” They proposed several mechanisms to explain vicarious pain, amongst which empathy or processes underlying empathy, hypervigilance to pain, chronic prior pain and trauma. According to this model, vicarious pain is a maladaptive form of empathic processing. Empathy has been defined in various ways, but generally features the capacity to understand and respond to the unique affective experiences of another person (Decety and Jackson, 2006). The role of empathy in vicarious pain experiences is yet unclear. In the study of Osborn and Derbyshire (2010), a group of pain responders and non-pain responders were subsequently matched for trait empathy Interpersonal Reactivity Index (IRI); consequently no differences occurred between both groups regarding this trait. Undergraduate students who reported an actual noxious somatic experience in response to images or clips depicting noxious events scored higher on a measure of state empathy than non-vicarious pain responders. Although the pain responders displayed more state empathy evoked by the images and movie clips, this was not correlated with reported pain intensity. However, in two recent studies, no differences were found between amputees with vicarious pain, amputees without vicarious pain responses, and non-amputee controls on measures of empathic ability (Giummarra et al., 2010; Fitzgibbon et al., 2012b).

Prior trauma may be the modulating variable inducing hypervigilance to pain cues, according to the model of Fitzgibbon et al. (2010b). Hypervigilance for pain is an over-alertness to pain-related information, and is installed when pain or anticipated pain becomes a current concern (Crombez et al., 2005). As such, vicarious pain may be an exaggerating response to the anticipation of observed pain (Giummarra et al., 2010; Fitzgibbon et al., 2012c). Therefore, we may expect that participants high in hypervigilance for pain report more vicarious pain experiences independent of any pre-existence of chronic (prior) pain. As yet, the proposed underlying mechanisms remain largely untested (Fitzgibbon et al., 2010b).

The primary aim of the present study is to develop an experimental paradigm allowing the measurement of vicarious pain experiences in people who explicitly report vicarious pain in daily life. A secondary aim was to explore the role of two potential moderators, i.e., dispositional empathy and hypervigilance for pain. To address these questions we developed a paradigm inspired by the work of Banissy and Ward (2007) on vicarious touch. In a first experiment, pre-selected undergraduate students reporting vicarious pain in daily life (i.e., “pain responders”) and a comparison group not reporting vicarious pain, were presented a series of videos showing hands being pricked, whilst receiving occasionally pricking experiences themselves in the same spatial location (congruent trials) or in the opposite location (incongruent trials) as the visual stimuli. Participants were instructed to report as rapidly as possible the spatial location of the administered somatosensory stimuli. First, we expected a higher frequency of vicarious pain during the experiment in the group reporting vicarious pain in daily life compared to the comparison group. In analogy with the study of Banissy and Ward (2007) in vicarious touch responders, we also expected that vicarious pain responders would be slower in incongruent relative to congruent trials. Second, we explored the effects and moderating role of dispositional empathy and hypervigilance to pain upon experiences of vicarious pain. In experiment 2, we aimed at replicating the findings of experiment 1, though with some procedural changes. Additionally, we explored the effect of the rubber hand illusion (RHI) upon vicarious pain, and differences between pain responders and controls in RHI experience. As pain responders experience bodily illusions in response to another in pain, we expect their experience of the rubber hand illusion to be more pronounced compared to controls.

## Experiment 1

### Method

#### Participants

Participants were recruited from a pool of approximately 682 undergraduate students from Ghent University who were invited to complete questionnaires screening for, amongst others, the experience of vicarious pain in daily life (November 2010 to January 2011). Specifically, participants were asked to indicate the extent to which they agreed with the question “Do you have the feeling experiencing pain when you observe another person in pain?” on a five point scale (0 = strongly disagree; 1 = disagree; 2 = neutral; 3 = agree; 4 = agree; 5 = strongly agree). This item was specifically developed for this study and was based upon the work of Banissy and colleagues (2009). Two-hundred fourteen students completed the screening questionnaires (31.38%). In line with Banissy and colleagues (2009), participants scoring 4 or higher (22.90%, *n* = 49) were invited to take part in the experiment. We also invited randomly 20 of those who scored 1 or lower. In total, thirty students (23 women, 7 men) agreed to participate. Mean age was 21.87 years (*SD* = 5.99, range: 18–49 years). All participants were Caucasian. Participants received either course credits for participation in this experiment (*n* = 13) or were paid (*n* = 17) 8 euro. Ethical approval was obtained from the Ethics committee of the Faculty of Psychology and Educational Sciences of Ghent University, Belgium.

#### Apparatus and stimuli

***Visual stimuli***. Visual stimuli consisted of 10 short videos with a duration of 3 s. Each video depicted a scene in which a left and right hand was presented, with one of the two hands being pricked by a sharp object (2000 ms after video onset). Five types of sharp objects were used across all videos, i.e., a safety pin, a needle, and three different syringes. Location of penetration (left vs. right hand) and type of sharp object were counterbalanced across videos. Videos were presented by INQUISIT Millisecond software (http://www.millisecond.com) on a Dell computer with a 19-in. CRT-monitor.

***Somatosensory stimuli***. Somatosensory stimuli were electrocutaneous stimuli (ES, bipolar, sinusoide, 200 Hz), delivered between thumb and index finger by two lubricated Medcat surface electrodes (1 cm diameter) of a constant current stimulator (DS5, Digitimer Ltd, Hertfordshire, UK). The duration of the ES was always 200 ms. The intensity of the electrocutaneous stimulus was individually determined. In a work up procedure, individuals were presented with stimuli of increasing intensity until a pricking sensation was reported. At the start the intensity was 0.25 mA, and increased by 0.25 mA for each next stimulus. Such procedure was performed for both the left and the right hand (used intensities: left: *M* = 0.78 mA, range: 0.25–1.5 mA; right: *M* = 0.75 mA, range: 0.25–1.5 mA).

#### Self report measures

To assess vicarious pain experiences in daily life, participants were asked to indicate the extent to which they agreed with the question “Do you have the feeling experiencing pain when you observe another person in pain?” on a five point scale (0 = strongly disagree; 5 = strongly agree). This question was used for the initial screening and read ministered during the lab experiment to classify participants in the pain responder group and the comparison group. At our university, the initial screening is anonymous and data from the screening can only be used to select participants but not for other research purposes.

Hypervigilance for pain was assessed by the Dutch version of the Pain Vigilance and Awareness Questionnaire (PVAQ; McCracken, 1997; Roelofs et al., 2003). This questionnaire consists of 16 items to be scored on a six-point scale (0 = never; 5 = always). The PVAQ consists of two subscales: attention to pain (e.g., ‘I pay close attention to pain’) and attention to changes in pain (e.g., ‘I am quick to notice changes in pain intensity’) (Roelofs et al., 2003). The questionnaire can be used in both clinical (McCracken, 1997; Roelofs et al., 2003) and non-clinical (McWilliams and Asmundson, 2001; Roelofs et al., 2002) samples. Higher scores are indicative of more vigilance to pain. The Dutch version of the PVAQ is reliable and valid (Roelofs et al., 2002, 2003). Cronbach's alpha for the present study was 0.89.

Dispositional empathy was assessed with the Dutch version of the (IRI; Davis, 1983; De Corte et al., 2007). The questionnaire contains 28 items and consists of 4 subscales: ‘Perspective Taking’ (i.e., cognitively taking the perspective of another, e.g., “I sometimes try to understand my friends better by imagining how things look from their perspective.”), ‘Fantasy’ (i.e., emotional identification with characters in books, films etc., e.g., “When I watch a good movie, I can very easily put myself in the place of a leading character.”), ‘Empathic Concern’ (i.e., feeling emotional concern for others, e.g., “I am often quite touched by things that I see happen.”) and ‘Personal Distress’ (i.e., negative feelings in response to the distress of others, e.g., “When I see someone who badly needs help in an emergency, I go to pieces.”). Each item is rated on a scale ranging from 1 (‘does not describe me very well’) to 5 (‘describes me very well’). This questionnaire has shown to be reliable and valid (Davis, 1983; De Corte et al., 2007). Cronbach's alpha's in the current study were 0.78 (fantasy scale), 0.61 (empathic concern), 0.79 (personal distress) and 0.39 (Perspective Taking). The latter subscale was omitted from the analyses because of the low reliability score.

Intensity and the (un)pleasantness of the electrocutaneous stimuli were rated on eleven-point numerical rating scales (0 = ‘not intense’; 10 = ‘intense’ respectively −5 = ‘unpleasant’; +5 = ‘pleasant’).

#### Procedure

***Preparation phase***. Participants were informed that they would feel stimuli, varying in intensity and length, on their left, right or both hands during the experiment. After signing the informed consent, a pair of electrodes was attached to each hand. The skin at the electrode sites was first abraded with a peeling cream (Nihon Kohden) in order to reduce skin resistance. Subsequently, the stimulus intensity level was established for each hand. Questions measuring the (un)pleasantness and intensity of the somatosensory stimulus were administered. Participants were seated in front of a table, at about 60 cm away from the computer screen and were informed that different videos would be presented which they needed to watch attentively. Hands of the participants were covered by means of a box and placed on the table in front of the screen. Participants were told that when a somatosensory stimulus was administered on both hands, the intensity could vary across hands and that also trials without any stimulus would be included. In reality, only one fixed predetermined intensity was applied for each hand.

**Figure 1:**
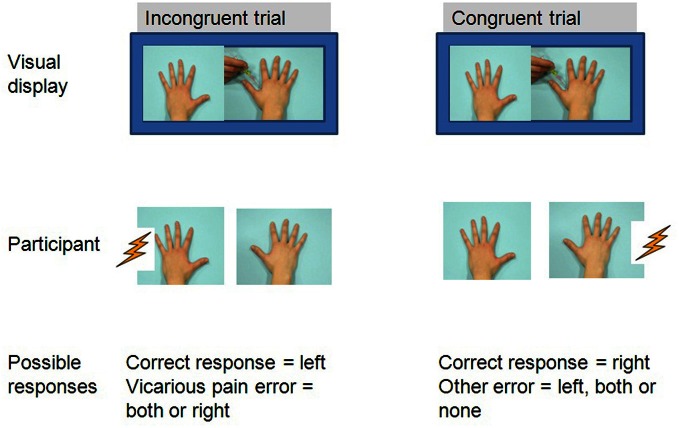
**Example of a possible trial**.

***Experiment phase***. Each trial began with a fixation cross (1000 ms duration) presented in the middle of the screen. Next, one of 10 different videos was presented. In two third of the trials, an electrocutaneous stimulus was delivered 2050 ms after video onset either on the left hand, the right hand, or on both hands of the participant. In line with Banissy and Ward (2007), the electrocutaneous stimulus was administered with a delay, which was 50 ms after the penetration of the sharp object in the observed hand. This resulted in the following trial types: (1) congruent trials, (2) incongruent trials, (3) trials in which no somatosensory stimuli were administered and (4) trials in which both hands of the participant received somatosensory stimuli. In congruent trials, somatosensory stimuli and visual stimuli were presented at the same spatial location (e.g., right). In incongruent trials, somatosensory stimuli and visual stimuli were presented in the opposite spatial location (e.g., left and right). The experiment started with 8 practice trials. The actual experiment phase consisted of three blocks of 64 trials, resulting in a total of 192 trials. There were 60 congruent trials, 60 incongruent trials, 60 trials without ES and 12 trials with ES at both hands equally divided over the three blocks. This latter trial type was added to make the response ‘both’ applicable and feasible. Visual stimuli were presented when ES was present or absent. Trial types were equally distributed across blocks. Order of trial types was randomized within each block. An overview of all trial types is presented in Table 1. During each trial, participants were requested to report whether a physical sensation was felt and indicate its location as quickly and accurately as possible by reporting aloud “left,” “right” or “both.” Reaction times were recorded by means of a voice key (see Figure 1). The experimenter coded the response by pressing the corresponding response button (left, right or both). The participant was instructed not to respond when no sensation was felt. In such situation a trial was considered completed when 2000 ms had elapsed after the video was ended. The completion of the experiment took approximately 50 min. Vicarious pain errors were calculated from incongruent trials and from trials in which no ES was administered. A vicarious pain error was considered present when participants reported feeling a pricking sensation in the same spatial location as the visual stimulus without the administration of an actual ES at that location.

**Table 1:** **An overview of all trial types (experiment 1 - experiment 2)**.

**Reported site**	**Congruent trials**				**Incongruent trials**				**No tactile stimulation**			
	**Correct site**	**Opposite site to visual and tactile**	**Both hands**	**No hands**	**Correct site**	**Opposite site (= visual site) *vicarious error***	**Both hands *vicarious error***	**No hands**	**Site congruent to visual *vicarious error***	**Opposite site to visual**	**Both hands**	**No hands**
**EXPERIMENT 1**												
%	93.27%	0.33%	2.07%	3.27%	90.40%	0.93%	3.00%	4.53%	1.40%	0.33%	0.20%	97.60%
**EXPERIMENT 2**												
%	94.00%	0.17%	0.42%	4.25%	92.00%	0.25%	1.42%	5.17%	0.67%	0.42%	0.00%	98.17%

Voice key errors are not included.

***Post-experiment phase***. After the experiment, participants were requested to fill out self-report scales measuring vicarious pain experiences in daily life, hypervigilance for pain (PVAQ) and empathic disposition (IRI).

#### Statistical analysis

Using the same criteria as during the screening, 14 participants were categorized in the pain responder group and 11 in the comparison group. Participants who did not fulfill these criteria at the moment of testing were excluded from analysis (*n* = 5).

To test the hypothesis that pain responders make more vicarious pain errors, count regression models were applied as the use of linear models is considered less appropriate when the frequency of responses has a skewed distribution that violates the normality assumption (e.g., Vives et al., 2006). Poisson regression is the basic model to analyze count data, but the variance of counts is often larger than the mean (overdispersion). The Negative Binomial (NB) regression, a Poisson regression with an overdispersion, may therefore better fit the data (e.g., Gardner et al., 1995). As count data may additionally exhibit a lot of zero counts, zero-inflated extensions of both models, called Zero-Inflated Poisson (ZIP) and Zero-Inflated NB (ZINB) models have been developed (see Karazsia and van Dulmen, 2010; Loeys et al., 2012). Deviance tests and Vuong test were used to select the best fitting count distribution for the dependent variable.

After the best fitting count model was chosen, several models were run. The first model contained the predictor ‘group’; the dependent variable was the number of vicarious pain errors. In subsequent analyses, participants' characteristics were added as second predictor in the model to explore whether PVAQ respectively IRI (subscales) had a moderating role.

Dummy coding was used for the categorical variables and standardized z-scores for the continuous predictors. Regression coefficients are exponentiated (*e*^*B*^) and called Rate Ratios (RRs). In percentages—100 × (*e*^*B*^ − 1)—RRs reflect the percentage decrease (RR < 1) or increase (RR > 1) in the expected frequency of vicarious pain errors for each standard deviation increase in the independent variable. R (version 2.15.1) was used to fit the count models.

To test whether participants in the pain responder group have higher hypervigilance and dispositional empathy scores compared with the comparison group, independent-samples *t*-tests were performed. To test whether pain responders show a larger congruency effect than non-pain responders (see Banissy and Ward, 2007), a 2 (congruency: congruent vs. incongruent) × 2 (group: comparison vs. pain responders) repeated measures ANOVA was performed, with congruency entered as within-subject variable and group as between-subject variable. Error trials and trials with responses faster than 200 ms or slower than 3 *SD* above the individual mean reaction time of each trial type were removed from RT analyses. These analyses were conducted with an α < 0.05, using SPSS statistical software, version 21.0 for Windows.

### Results

#### Descriptive statistics

Mean scores, standard deviations and correlations of experiment 1 are presented in Tables 2, 3. Because the variable (un)pleasantness did not have a normal distribution, Spearman correlations were computed for this particular variable (Kolmogorov-Smirnoff, *p* < 0.05). Mean age was 21.50 years in the pain responder group (*SD* = 4.16, range: 18–34 years) and 23.27 years (*SD* = 8.76, range: 18–49 years) in the comparison group. Of all participants, 27.3% indicated to have experienced an episode of chronic pain during their life (pain duration longer than 3 months). There was no significant difference between both groups [*t*_(20)_ = −1.16, *p* = 0.26]. In 2.7% of the incongruent trials and trials without any ES, vicarious pain errors were made (80 vicarious pain errors from a total of 3000 trials), mainly in the pain responder group (83.75% of all vicarious pain errors; *n* = 67). Two participants in the pain responder group were responsible for 66.25% of all vicarious pain errors (53 of a total of 80 vicarious pain errors). The number of vicarious pain errors did not differ across the 3 blocks (Kruskal-Wallis, *p* = 0.12). No difference was found between both groups in PVAQ scores [*t*_(23)_ = −1.93, *p* = 0.07] or empathy scores (subscales all *p* ≥ 0.10).

**Table 2:** **Mean scores and standard deviations of all measures (study 1)**.

	**M (SD) pain responder group**	**M (SD) comparison group**	**M (SD) total group**
1. RT incongruent trials	784.48 (118.44)	674.45 (74.34)	736.07 (114.06)
2. RT congruent trials	719.79 (136.86)	628.82 (70.88)	679.76 (119.84)
3. Intensity (0–10)	4.46 (1.66)	4.77 (1.65)	4.6 (1.63)
4. (Un)pleasantness	−1.43 (1.41)	−1.95 (0.76)	−1.66 (1.18)
5. PVAQ	39.62 (13.64)	30.0 (10.52)	35.39 (13.06)
6. EC	19.21 (3.38)	17.91 (3.75)	18.64 (3.53)
7. FS	21.29 (4.46)	19.00 (4.77)	20.28 (4.65)
8. PD	12.50 (6.16)	15.82 (3.34)	13.96 (5.30)

PVAQ, Pain Vigilance and Awareness Questionnaire; EC, Empathic Concern; FS, Fantasy Scale; PD, Personal Distress; RT, Reaction times.

**Table 3:** **Pearson/spearman correlations of all measures (study 1)**.

	**2**	**3**	**4**	**5**	**6**	**7**	**8**
1. RT incongruent trials	0.91^**^	−0.17	−0.05	0.03	−0.23	0.17	−0.51^**^
2. RT congruent trials	−	−0.24	−0.02	0.01	−0.32	0.09	−0.57^**^
3. Intensity (0–10)		−	−0.62^**^	0.41^*^	0.12	0.26	0.53^**^
4. (Un)pleasantness			−	−0.41^*^	0.22	−0.43^*^	−0.24
5. PVAQ				−	0.13	0.18	−0.07
6. EC					−	0.41^*^	0.17
7. FS						−	0.04
8. PD							−

PVAQ, Pain Vigilance and Awareness Questionnaire; EC, Empathic Concern; FS, Fantasy Scale; PD, Personal Distress; RT, Reaction times.

* p < 0.05;

** p < 0.01.

#### Vicarious pain errors

The NB model was found to be the best fitting count model (χ^2^ [1, *N* = 25] = 149.26, *p* < 0.001; V = −1.33, *p* = 0.09) to test the influence of group (pain responder vs. comparison group) upon the number of vicarious pain errors. In a first step, group was added as a predictor. Results showed that the number of vicarious pain errors significantly raised with 305% (RR = 4.05, *p* = 0.04; [95% CI: −0.02, 2.78]) when participants reported vicarious pain experiences in daily life (pain responder group) compared to the comparison group.

**Figure 2:**
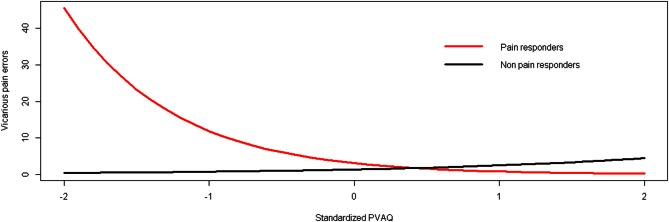
**The relationship between hypervigilance for pain (PVAQ) and vicarious pain errors as moderated by group (study 1)**.

In order to explore the moderating role of individual differences in hypervigilance for pain (PVAQ) and dispositional empathy (IRI), additional models were run with PVAQ or IRI subscales entered as a second predictor and in interaction with group. A significant interaction was found between group and PVAQ (*p* < 0.01; [95% CI: −3.40, −0.57]). For pain responders, the probability of making vicarious pain errors decreased by 74% (RR = 0.26) for every standard deviation increase in hypervigilance for pain. For the comparison group, the probability of making vicarious pain errors increased by 79% (RR = 1.79) for every standard deviation increase in hypervigilance for pain (Figure 2). No main effect of hypervigilance for pain was found (*p* = 0.28).

Furthermore, no interaction was found between group and subscales ‘fantasy’ (*p* = 0.22), ‘personal distress’ (*p* = 0.99) and ‘empathic concern’ (*p* = 0.61). Also no main effects of these subscales were found (all *p* > 0.44).

#### Reaction times

A 2 (congruency: congruent vs. incongruent) × 2 (group: comparison vs. pain responder group) repeated measures ANOVA showed a main effect of group. In particular, the pain responder group was slower in both congruent and incongruent trials compared to the comparison group [*F*_(1, 23)_ = 5.70, *p* = 0.03]. Furthermore, also a main effect of congruency was observed [*F*_(1, 23)_ = 29.84, *p* < 0.01] indicating that all participants were faster on congruent than on incongruent trials. Contrary to expectations, no interaction was found between congruency and group [*F*_(1, 23)_ = 0.89, *p* = 0.36].

### Discussion

Current results indicate that our paradigm allows us to measure vicarious pain experiences in healthy students and revealed only a small percentage of vicarious pain errors. As the sample size of the first experiment was relatively small, a second experiment was performed to test whether the results could be replicated. Furthermore, a more stringent recruitment procedure was used than in experiment 1 where vicarious pain experiences in daily life were measured by means of only one item. As pain responders experience bodily illusions in response to viewing another's pain, an additional aim of the second experiment was to explore whether pain responders report a stronger rubber hand illusion experience than controls (Botvinick and Cohen, 1998). Finally, we also investigated whether the rubber hand illusion experience was related to participants' vicarious pain errors.

## Experiment 2

### Methods

#### Participants

Participants were recruited from a pool of approximately 647 undergraduate students from Ghent University who were invited to complete several questionnaires (October to November 2011). One of these questionnaires intended to assess the experience of vicarious pain experiences in daily life by means of four items adapted from Banissy et al. (2009). Participants were asked to indicate on an eleven point scale (0–10; totally disagree—totally agree) the extent to which they agreed with the questions: “Do you feel pain in your own body when you see someone accidently bump against the corner of a table?”, “Do you have the feeling experiencing pain when you observe another person in pain?”, “Do you feel bodily pain when you observe another person in pain?” and “Do you feel a physical sensation (e.g., tingling, stabbing, …) when you observe another person in pain?”. Completed questionnaires were available from 348 students (53.79%). As no standard cut-off for the presence of vicarious pain was available, we invited all participants who scored ≥ 6 on all questions (6.61%, *n* = 23). This cut-off preserves a balance between extreme values (inviting the highest scoring vicarious pain responders) and a minimum of pain responders to participate. We also invited randomly 20 of those who scored ≤ 1 on all questions.

In total, 24 undergraduates (23 women) agreed to participate. Their mean age was 19.17 years (*SD* = 1.81, range: 17–23 years). All participants, except one, were Caucasian. Participants received either course credits for participation in this experiment (*n* = 21) or were paid (*n* = 3) 8 euro. Ethical approval was obtained from the Ethics committee of the Faculty of Psychology and Educational Sciences of Ghent University (Belgium).

#### Design, apparatus and stimuli

The design, apparatus and stimuli, were similar as in experiment 1. The mean intensity of the somatosensory stimuli was 0.74 mA (range: 0.50–1 mA) for the left hand and 0.69 mA (range: 0.50–1mA) for the right hand.

#### Self-report measures

To assess vicarious experiences in daily life, participants were asked to indicate on an 11-point scale (0–10; totally disagree—totally agree) the extent to which they agreed with each of the four items, which were also used in the initial screening. This questionnaire was readministered during the procedure in the lab as the first screening was anonymous. Cronbach's alpha in the current study was 0.97.

Hypervigilance to pain (PVAQ; Cronbach's α = 0.91) and empathic disposition (IRI; fantasy scale Cronbach's α = 0.84, empathic concern Cronbach's α = 0.69, personal distress Cronbach's α = 0.77, perspective taking, Cronbach's α = 0.39) were assessed in the same way as in experiment 1. As in experiment 1, the perspective taking subscale was omitted from the analyses because of the low reliability score.

Rubber hand illusion (RHI) experience was measured by means of nine items (e.g., ‘It felt as if the rubber hand was my hand’; Botvinick and Cohen, 1998). Participants indicated the extent to which they agreed or disagreed on a 15 cm scale. Seven positions were marked ranging from strongly disagree (—) to strongly agree (+++). A total score for the RHI experience was based upon the sum score of all items (Cronbach's α = 0.79).

#### Procedure

The first part of the procedure used in this experiment was identical to the applied procedure in experiment 1. Subsequent to the experiment, participants took part in a rubber hand illusion (RHI) test. The test was set up and conducted in line with previous RHI studies (Botvinick and Cohen, 1998). Participants were seated with their both arms placed upon a table. Their right hand was positioned next to a screen, outside the view of the participant. A right-handed life-sized rubber hand was placed on the table directly in front of the subject with its index finger 20 cm to the right of the participant's index finger. A black cape extending from their neck to the table obscured the view of their upper arms throughout the experiment. Participants were asked to focus on the rubber hand. Two small paintbrushes were used to stroke the participant's and rubber hand's index fingers during 3 min, synchronizing the timing of the brushing as closely as possible. After the RHI test, participants were requested to fill in a short questionnaire about their experience during the RHI test (see Botvinick and Cohen, 1998).

#### Statistical analysis

Participants were categorized in a pain responder group and a comparison group based upon the sum of their responses on the items measuring vicarious pain in daily life, administered during the experiment. As no cut-off was available, we considered to maintain all participants whose sum score was <15 (*n* = 7; comparison group) and those whose sum score was >25 (*n* = 13; pain responder group) as this cut-off preserves a balance between extreme values (the most extreme scoring vicarious pain responders) and a minimum of pain responders to analyze. Four participants scoring between 15 and 25 were excluded from the analyses.

To test the hypothesis that pain responders make more vicarious pain errors, we applied similar statistical analyses as those performed in experiment 1. Additional analyses were performed related to RHI. To investigate whether pain responders had a higher score on the questions measuring the RHI than the comparison group, we used a one sample *t*-test. We also explored whether the RHI experience was related to the number of vicarious pain errors in the behavioral paradigm.

### Results

#### Descriptive statistics

Mean scores, standard deviations and correlations for the second experiment are presented in Tables 4, 5. The variables intensity and empathic concern did not have a normal distribution, therefore spearman correlations are indicated for these particular variables (Kolmogorov-Smirnoff, *p* < 0.05). The mean age of the participants in the pain responder group was 19.85 years (*SD* = 2.03, range: 18–23) and 18.29 years for the comparison group (*SD* = 1.25, range: 17–21 years). Of all participants, 52.6% indicated to have experienced an episode of chronic pain during their life (pain duration longer than 3 months). This was not significantly different between both groups [*t*_(17)_ = −0.62, *p* = 0.54].

**Table 4:** **Mean scores and standard deviations (study 2)**.

	**M (SD) pain responder group**	**M (SD) comparison group**	**M (SD) total group**
1. RT incongruent trials	711.07 (155.00)	685.51 (86.72)	702.12 (133.06)
2. RT congruent trials	681.10 (150.37)	651.05 (58.46)	670.59 (124.80)
3. Intensity	4.38 (2.31)	3.86 (2.46)	4.20 (2.31)
4. (Un)pleasantness	−1.81 (1.16)	−1.5 (1.08)	−1.70 (1.12)
5. PVAQ	42.23 (14.14)	42.00 (9.13)	42.15 (12.36)
6. EC	21.62 (2.02)	18 (4.58)	20.35 (3.51)
7. FS	20.85 (4.63)	19.57 (5.86)	20.40 (4.98)
8. PD	14.54 (4.99)	14.43 (5.22)	14.50 (4.94)
9. RHI	753.77 (206.04)	631.86 (199.73)	711.10 (207.29)

PVAQ, Pain Vigilance and Awareness Questionnaire; EC, Empathic Concern; FS, Fantasy Scale; PD, Personal Distress; RHI, Rubber Hand Illusion; RT, Reaction times.

**Table 5:** **Pearson/spearman correlations of all measures (study 2)**.

	**2**	**3**	**4**	**5**	**6**	**7**	**8**	**9**
1. RT incongruent trials	0.96^**^	−0.16	0.06	0.27	−0.12	0.18	−0.11	0.24
2. RT congruent trials	−	−0.07	0.10	0.33	−0.14	0.18	−0.10	0.23
3. Intensity		−	−0.61^**^	0.10	−0.18	0.17	0.24	0.16
4. (Un)pleasantness			−	0.01	0.02	−0.07	−0.21	0.11
5. PVAQ				−	0.28	0.23	0.47^*^	0.48^*^
6. EC					−	0.28	0.14	0.12
7. FS						−	0.06	0.39
8. PD							−	0.46^*^
9. RHI								−

PVAQ, Pain Vigilance and Awareness Questionnaire; EC, Empathic Concern; FS, Fantasy Scale; PD, Personal Distress; RHI, Rubber Hand Illusion; RT, Reaction times.

* p < 0.05;

** p < 0.01.

In 0.88% of the trials, vicarious pain errors were made (21 vicarious pain errors from a total of 2400 trials), especially in the pain responder group (90.48% of all vicarious pain errors, *n* = 19). Three pain responders were responsible for 76.19% of all vicarious pain errors (16 of a total of 21 vicarious pain errors). The number of vicarious pain errors did not differ across the three blocks (Kruskal-Wallis, *p* = 0.75). Furthermore, no significant difference was found between the pain responder group and the comparison group concerning the rubber hand illusion experience [*t*_(18)_ = −1.28, *p* = 0.22]. Also no differences were found between both groups regarding dispositional empathy scores (all *p* ≥ 0.60) and hypervigilance for pain [*t*_(18)_ = −0.04, *p* = 0.97].

#### Vicarious pain errors

To investigate the impact of group (comparison vs. pain responder group) upon the number of vicarious pain errors, the NB-model was chosen as count model (χ^2^ [1, *n* = 20] = 27.84, *p* < 0.001; V = 1.71, *p* = 0.24). The results of the NB regression testing showed that group did not influence the frequency of vicarious pain errors (*p* = 0.17).

In subsequent analyses, several models were run containing observer's characteristics such as PVAQ, subscales of the IRI and rubber hand illusion as a second predictor in the interaction to explore a moderating role. PVAQ did not significantly interact with group (*p* = 0.86), nor did the fantasy scale (*p* = 0.44), personal distress (*p* = 0.55), or rubber hand illusion (*p* = 0.39). Also no main effect was found of the PVAQ (*p* = 0.57), nor of the different subscales of the IRI (all *p* > 0.24) or RHI (*p* = 0.34).

#### Reaction times

A 2 (congruency: congruent vs. incongruent) × 2 (group: comparison vs. pain responder group) repeated measures ANOVA revealed no main effect of group; indicating that pain responders were not slower compared to the comparison group [*F*_(1, 18)_ = 0.21, *p* = 0.66]. Results did however reveal a main effect of congruency [*F*_(1, 18)_ = 13.73, *p* = 0.002], indicating that participants in general were faster on congruent than on incongruent trials. No interaction was found between congruency and group [*F*_(1, 18)_ = 0.07, *p* = 0.80].

### Discussion

**Figure 3:**
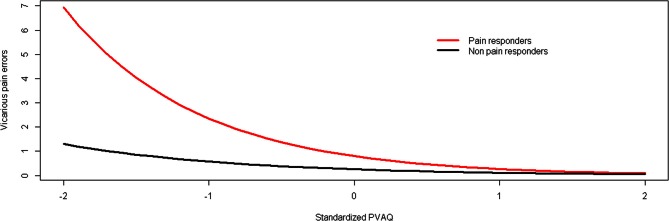
**The relationship between hypervigilance for pain (PVAQ) and vicarious pain errors as moderated by group (study 2)**.

In contrast to experiment 1, individuals reporting vicarious pain experiences in daily life did not report more vicarious pain errors in our behavioral paradigm than individuals from the comparison group. Although a negative association was observed between the number of vicarious pain errors and hypervigilance for pain in the pain responder group (see Figure 3), this effect proved to be non-significant. This may be due to a low sample size (*n* = 20). In that respect, it may however be that the results of both studies do not differ (Schmidt, 2010). To explore this issue further, we performed an analysis of the data combined from both experiments, and added an extra between-subject variable study (experiment 1 vs. 2).

## Overall analyses

### Results

#### Descriptive results

Mean scores, standard deviations and correlations of the pooled data are presented in Tables 6, 7. As the congruent and incongruent RT as well as the self-report variables intensity, (un)pleasantness, personal distress and fantasy scale were not normally distributed (Kolmogorov-Smirnoff, *p* < 0.05) we reported Spearman correlations for these variables. To test whether both groups differed in hypervigilance and empathic concern, independent-sample t-tests were performed. Participants in the pain responder group were more empathic concerned compared to participants in the comparison group [*t*_(43)_ = −2.33, *p* = 0.03]. No difference was found between both groups in hypervigilance for pain [*t*_(43)_ = −1.59, *p* = 0.12]. For all analyses regarding reaction times, log10 transformation was used to normalize data.

**Table 6:** **Mean scores and standard deviations (overall analyses)**.

	**M (SD) pain responder group**	**M (SD) comparison group**	**M (SD) total group**
1. RT incongruent trials	749.14 (139.64)	678.76 (77.04)	720.98 (122.60)
2. RT congruent trials	701.16 (142.09)	637.47 (65.47)	675.69 (120.75)
3. intensity	4.43 (1.96)	4.42 (1.99)	4.42 (1.95)
4. (Un)pleasantness	−1.61 (1.29)	−1.78 (0.89)	−1.68 (1.14)
5. PVAQ	40.88 (13.68)	34.67 (11.43)	38.39 (13.06)
6. EC	20.37 (3.01)	17.94 (3.96)	19.40 (3.59)
7. FS	21.07 (4.46)	19.22 (5.06)	20.33 (4.74)
8. PD	13.48 (5.62)	15.28 (4.08)	14.20 (5.09)

PVAQ, Pain Vigilance and Awareness Questionnaire; EC, Empathic Concern; FS, Fantasy Scale; PD, Personal Distress; RT, Reaction times.

**Table 7:** **Pearson/Spearman correlations of all measures (overall analyses)**.

	**2**	**3**	**4**	**5**	**6**	**7**	**8**
1. RT incongruent trials	0.89^**^	−0.14	0	−0.14	−0.21	0.19	−0.37^*^
2. RT congruent trials	−	−0.13	−0.01	−0.15	−0.20	0.18	−0.44^**^
3. intensity		−	−0.68^**^	0.21	0.02	0.15	0.40^**^
4. (Un)pleasantness			−	−0.22	0.07	−0.28	−0.22
5. PVAQ				−	0.22	0.20	0.16
6. EC					−	0.38^*^	0.21
7. FS						−	−0.02
8. PD							−

PVAQ, Pain Vigilance and Awareness Questionnaire; EC, Empathic Concern; FS, Fantasy Scale; PD, Personal Distress; RT, Reaction times.

* p < 0.05;

** p < 0.01.

#### Vicarious pain errors

To investigate the impact of group (pain responder vs. comparison group) upon the number of vicarious pain errors, the NB-model was again selected as best fitting count model (χ^2^ [1, *n* = 45] = 198.34, *p* < 0.001; *V* = −0.55, *p* = 0.29). First, we checked whether study (experiment 1 vs. 2) had an impact upon number of vicarious pain errors. The relation between the number of vicarious pain errors and PVAQ (*p* = 0.66) and group (*p* = 0.86) was not dependent upon study (1 vs. 2). Also the interaction between the number of vicarious pain errors and study × group (*p* = 0.33) was not significant. Only a marginal main effect of study was observed, suggesting a slightly higher prevalence of vicarious pain errors in the first study (*p* = 0.06). No interactions of study with any of the independent variables were found. To test whether pain responders make more vicarious pain errors compared to non-pain responders, group was added as a single predictor. The number of vicarious pain errors significantly raised with 282% (RR = 3.82, *p* = 0.03; [95% CI: 0.09, 2.54]) when participants reported vicarious pain in daily life (pain responder group) compared with the comparison group.

Additional analyses were run containing observer's characteristics such as PVAQ or subscales of the IRI as a second predictor in interaction with group to explore a possible moderating role. A significant interaction was observed between group and PVAQ (*p* = 0.02; [95% CI: −2.52, −0.05]). The size of the RR (0.96) demonstrated that the probability of making vicarious pain errors for the non-pain responders decreased by 4% for every standard deviation increase in hypervigilance for pain. For the pain responders, the probability of making vicarious pain errors decreased by 73% (RR = 0.27) for every standard deviation increase in hypervigilance for pain. The subscales of the IRI did not significantly interact with group (‘fantasy scale,’ *p* = 0.26; ‘empathic concern,’ *p* = 0.68; ‘personal distress,’ *p* = 0.90).

#### Reaction times

A 2 (congruency: congruent vs. incongruent) × 2 (group: pain responders vs. comparison) × 2 (study: first vs. second study) repeated measures ANOVA showed no main effect for group [*F*_(1, 41)_ = 2.49, *p* = 0.12] and for study [*F*_(1, 41)_ = 0.30, *p* = 0.59]. Overall, participants were faster on congruent than on incongruent trials [*F*_(1, 41)_ = 39.60, *p* < 0.001]. In contrast with expectations, no interaction was found between congruency and group [*F*_(1, 41)_ = 0.16, *p* = 0.69].

## General discussion

Two experiments are reported, in which an experimental paradigm was used to assess the presence of vicarious pain experiences in healthy participants. Additionally, we explored the effects of some potential moderators proposed by Fitzgibbon et al. (2010b), i.e., dispositional empathy, hypervigilance to pain and also the tendency to experience the rubber hand illusion. In both studies, undergraduates were categorized in a pain responder group and a comparison group based upon reported vicarious pain experiences in daily life. They were presented a series of videos showing hands being pricked whilst receiving occasionally painful pricking sensations (electrocutaneous stimuli) themselves. In congruent trials, pricking stimuli and visual stimuli were applied to the same spatial location (e.g., right). In incongruent trials, pricking stimuli and visual stimuli were in the opposite spatial location (e.g., left and right). Participants were required to report as fast as possible where they felt a pricking sensation.

The main results can be readily summarized. In experiment 1, we found that the used paradigm was sensitive to measure vicarious pain experiences in healthy students. Findings indicated that participants who reported vicarious pain experiences in daily life made more vicarious pain errors during the experiment than participants of the comparison group. Furthermore, the probability of making vicarious pain errors decreased steeply for the pain responder group when they showed an increased level of hypervigilance for pain, whereas the probability of making vicarious pain errors increased for the comparison group when they showed an increased level of hypervigilance for pain. In experiment 2, however, findings of experiment 1 were not confirmed. No influence was found of the group to which participants belonged on the number of vicarious pain errors made during the experiment. Also no relationship was found between the level of hypervigilance for pain and the number of vicarious pain errors made. There was also no relationship between the number of vicarious pain errors and the rubber hand illusion experience. In order to explore the possible difference between both experiments, we opted to merge the data of both experiments. Results of these analysis showed that there was no difference in both experiments related to the findings. The overall results (i.e., of the merged data) were in line with findings of experiment 1 and indicated that (1) participants who reported vicarious pain experiences in daily life made more vicarious pain errors during the experiment than participants of the comparison group and (2) the probability of making vicarious pain errors decreased steeply for the pain responder group when they showed an increased level of hypervigilance for pain, while vicarious pain errors showed only a little decrease in the comparison group. For reasons of clarity, the discussion will mainly focus upon the combined findings.

First, our study reveals that undergraduates report vicarious pain experiences in daily life, albeit that the prevalence of pain responders was low. In experiment 1, the prevalence was 22.9%. In experiment 2, it was 6.61%. The difference in prevalence of self-reported vicarious pain experiences in daily life between both experiments is probably due to the use of a more stringent cut-off to categorize pain and non-pain responders compared to Experiment 1. Overall, the prevalence of vicarious pain found in the current study is low in comparison with the prevalence reported by Osborn and Derbyshire (2010), which was almost 30%. One reason for this difference may relate to the fact that the prevalence number in the present study was based upon self-report of vicarious pain experiences in daily life whereas the prevalence number reported by Osborn and Derbyshire (2010) was based upon report of participants who were shown images of people perceiving pain. It is worthwhile for future studies to combine both approaches and to recruit people based upon questions measuring vicarious pain in combination with showing participants video clips of painful situations to check whether they are feeling pain experiences. The variability in prevalence illustrates the need to have clear criteria to identify pain responders in future research.

Second, overall the experimental paradigm was successful in eliciting vicarious experiences of pain, in particular in those reporting vicarious pain experiences in daily life. The number of vicarious pain errors doubled in participants reporting vicarious pain in daily life (i.e., pain responder group) compared to the comparison group. However, it should be noted that the total number of vicarious pain errors was low, and only a few participants from the pain responders group accounted for the phenomenon. Future research may focus upon these few pain responders and investigate on which variables they differ from other participants. First, the low number of vicarious pain errors could be due to the fact that felt and seen stimuli may result in a different sensation. Indeed, it might be that the sensation experienced by the electrocutaneous stimulus differs too much from the sensation experienced when being confronted with images of a pricking sensation. Indeed, the more actual somatosensory sensations are alike to the vicarious experiences, the more vicarious errors may occur in our experimental paradigm. This may however only be achieved with vague somatosensory stimuli of low intensity. Interestingly, in the study of Osborn and Derbyshire (2010), the most frequent descriptor that was selected from the McGill Pain Questionnaire to describe vicarious pain was “tingling.” Therefore, it would be interesting for future research to use tingling stimuli of a low intensity instead of electrocutaneous stimuli to investigate vicarious experiences. In line with this, pain responders in the study of Osborn and Derbyshire (2010) rated the average vicarious pain across all images rather low on a visual analogue scale (*M* = 1.9, *SD* = 2.4) ranging from 0 (no pain) to 10 (most pain imaginable). The experience of vicarious pain was dependent upon the content of the picture. In our study, the intensity of the ES were not rated as highly painful, since intensity ratings were on average around 4.4 on a 10-point scale (0 = not intense and 10 = intense), and unpleasantness ratings were on average −1.6 (−5 “unpleasant”; +5 “pleasant”). Our aim was to provide somatosensory stimuli that were not too painful and which induced experiences that were alike to the shown pricks. If somatosensory stimuli would be experienced too intense, it would be very easy to distinguish vicarious experiences from administered ES. With more intense ES, our prediction would be that no vicarious errors would occur. We included video clips showing hands being pricked. These videos depict less intense pain compared to the images and movies used in the study of Osborn and Derbyshire (2010). Vicarious pain may be elicited more easily when very intense pain is observed. The fact that pain responders in this study already experience vicarious pain during the mere observation of a subtle injury such as a needle prick is therefore very informative and interesting.

We explored the (moderating) role of several individual difference variables such as dispositional empathy, hypervigilance for pain and the degree to which the rubber hand illusion was experienced upon vicarious pain. Current findings do not provide support for the moderating role of dispositional empathy. Although the pain responder group was more empathic concerned, this had no influence upon the occurrence of vicarious pain errors. It might however be that, although dispositional empathy may not play a role as underlying mechanism in normal subjects reporting vicarious pain experiences, it might have an impact in individuals with prior chronic pain or trauma such as amputees, where vicarious experiences of pain are often experienced as more intense (Giummarra and Bradshaw, 2008; Fitzgibbon et al., 2010a). Also the degree in which the rubber hand illusion was experienced was not different for both groups. It had also no explanatory role in the experience of vicarious pain errors. In line with the model provided by Fitzgibbon and colleagues (2010b), we also explored whether the occurrence of vicarious pain errors was influenced by the degree of hypervigilance for pain. According to the theory of Fitzgibbon et al. (2010b), we expected pain hypervigilance to facilitate the production of vicarious pain errors as we expected pain responders to be overattentive to pain cues. As such, vicarious pain may be an exaggerating response to the anticipation of observed pain. Contrary to our expectations, more hypervigilance for pain was related to less vicarious pain errors in the group of pain responders, suggesting that hypervigilant participants were less misled by the visual stimuli. The same, albeit small, negative relation was found for the non-responder group. A possible explanation for this unexpected finding may relate to the fact that pain responders who are more focused upon the detection of somatic sensations experience less vicarious pain experiences. It is however unclear why hypervigilance for pain has a moderating role in making vicarious pain errors and how exactly this observer's characteristic prevents pain responders to make vicarious pain errors.

Taken all the literature together, there is preliminary evidence for vicarious pain experiences in response to observing pain in others (Fitzgibbon et al., 2010b). Until now there is little empirical investigation into this phenomenon. To date, the preliminary evidence regarding vicarious pain is primarily based upon anecdotal reports, and research in clinical populations with prior pain or trauma. Only little research is available on the conditions in which vicarious pain occurs and on the underlying mechanisms. Especially the role of empathy or processes underlying empathy have predominantly been investigated (e.g., Fitzgibbon et al., 2012a,b).

This study is one of the first to measure whether observers can feel pain themselves by observing pain in another individual measured by means of an experimental design. Insight into the conditions wherein pain is elicited by mere observation is of major significance for both the theory about pain as a biopsychosocial phenomenon and clinical practice. Theoretically, insight into the conditions and processes of vicarious pain is expected to fundamentally change the view about how pain is processed in the brain, demonstrating the important role of psychosocial variables (e.g., empathy, hypervigilance for pain), not only in the modulation (Van Damme et al., 2010) but also as cause of pain experiences in clinical and non-clinical populations. Further research is needed to investigate the underlying mechanisms of vicarious pain in a general population and in chronic pain patients. Also research is needed about the quality and intensity of the reported vicarious pain experiences and the difference between the reported vicarious experiences and the visual triggers (i.e., pain in another). Besides the neuro-imaging and behavioral research, it would be interesting to explore whether vicarious pain experiences are also reflected in different patterns regarding psychophysiological measures (e.g., heart rate, skin conductance). Other possibilities are to show more intense painful images to enhance chances for vicarious pain errors to occur. Other studies have suggested that empathic responses are substantially influenced by whether or not one attends to the feelings of the target through the explicit imagination of the target's feelings (Jackson et al., 2006; Preston et al., 2007; Fan and Han, 2008). Future research may therefore consider using not only real life images and movies but also specific instructions to manipulate participants' empathic responses to investigate whether this impacts the occurrence of vicarious experiences.

A number of limitations deserve further consideration, each of which point to directions for future research. First, only few people reported vicarious pain experiences in daily life, resulting in a small sample size in these experiments. We tried to overcome this by additional analyses of the pooled data of the two experiments. Although sample sizes were small, the amount of pain responders who took part in the experiments were comparable to other studies who included participants reporting vicarious bodily sensations (Banissy and Ward, 2007; Osborn and Derbyshire, 2010). Second, for the second experiment, different cut-offs were used for initial screening and during the lab experiment to classify participants in the pain responder group and the comparison group to preserve a minimum of pain responders to analyze. This implies that participants scored the different questions not exactly the same over time. As the initial screening is anonymous at our university, data from the initial screening is not linked to specific individuals, which makes it impossible to compare both ratings in each individual. Future research is needed to investigate the reliability and stability of this phenomenon across time.

## Conclusion

This new behavioral paradigm allowed measuring vicarious pain experiences in undergraduates. Vicarious pain experiences were found to be a rather rare phenomenon, elicited in only a subsample of participants reporting vicarious pain experiences in daily life. This behavioral paradigm is promising to investigate other underlying mechanisms (i.e., prior pain) of vicarious experiences of pain.

### Conflict of interest statement

The authors declare that the research was conducted in the absence of any commercial or financial relationships that could be construed as a potential conflict of interest.
